# Pulmonary Capillary Confinement Shapes Bacteria-engaging Neutrophil Fate Transition

**DOI:** 10.21203/rs.3.rs-10269644/v1

**Published:** 2026-07-22

**Authors:** Yoshikazu Tsukasaki, Kaori Masuhara

**Affiliations:** Louisiana State University Health Sciences Center at Shreveport; Louisiana State University Health Sciences Center at Shreveport

## Abstract

Neutrophils must deform to traverse pulmonary capillaries, yet whether this mechanical confinement shapes their inflammatory state is unclear. Quantitative lung intravital imaging shows that capillary transit is associated with GsMTx4-sensitive Ca^2+^ signals, PAD-linked nuclear remodeling and the emergence of motile anuclear neutrophil-derived cytoplasts. These cytoplasts preferentially associate with Pseudomonas aeruginosa in vivo, suggesting that the pulmonary capillary bed functions as a mechanical checkpoint for early host–microbe interactions.

The lung is continuously exposed to environmental microbes and particulates, and polymorphonuclear neutrophils (PMNs) act as major phagocytes in early host defense^1,2^. Because pulmonary capillaries are similar to, or smaller than, the diameter of circulating PMNs, these cells must repeatedly deform during capillary transit and transiently accumulate within the lung microvasculature as a marginated intravascular pool^3^. Although this pool is thought to support rapid immune surveillance, it can also contribute to microvascular injury during inflammation^2,4^. Whether pulmonary capillary confinement is merely a passive physical constraint on PMN passage or instead actively instructs PMN signaling state and host–microbe interactions has remained unclear.

To examine whether confinement in pulmonary capillaries is linked to PMN-intrinsic signaling in the inflamed lung, we performed quantitative two-photon intravital Ca^2+^ imaging in breathing mice using a computer-vision-based motion-correction approach (CoVSTii)^5,6^. In PMN-specific Salsa6f reporter mice^7,8^ imaged 1 h after lipopolysaccharide (LPS) challenge, PMNs rapidly accumulated within pulmonary capillaries and showed transient increases in intracellular Ca^2+^ during capillary transit (Fig. 1a, b and **Extended Data Movie 1**). Treatment with the mechanosensitive channel inhibitor GsMTx4 reduced the number of PMNs detected within the field of view and lowered both mean Ca^2+^ signal intensity and Ca^2+^ signal variance in individual PMNs (Fig. 1b–d). Single-cell tracking showed that Ca^2+^ intensity strongly correlated with cell perimeter, which we used as a surrogate for deformation during passage through narrow capillary segments, and that this coupling was lost with GsMTx4 treatment (Fig. 1e–g). These observations identify inflamed pulmonary capillary transit as an in vivo setting in which PMN deformation is coupled to GsMTx4-sensitive Ca^2+^ signaling, extending previous evidence that mechanical stimulation of PMNs can activate Ca^2+^-dependent antimicrobial responses^9,10^.

We next asked whether this signaling state is coupled to nuclear remodeling. To visualize nuclear dynamics in vivo, we administered the cell-permeable nuclear dye SYTO40 intravenously and imaged pulmonary PMNs 3 h after LPS challenge. At this time point, approximately 30–40% of PMNs lacked a detectable nuclear signal, consistent with anuclear PMN-derived cytoplasts (PMN_cyt_s)^11^ (Fig. 1h, k and **Extended Data Movie 2**). Inhibition of peptidylarginine deiminase (PAD) activity, which promotes histone citrullination and nuclear remodeling^12^, with Cl-amidine or inhibition of mechanosensitive ion channels with GsMTx4 shifted the multilobulated nuclear morphology of nucleated PMNs towards a more compact morphology, reflected by increased nuclear solidity, and significantly reduced PMN_cyt_ formation (Fig. 1h–l). Morphometric analysis further showed that, whereas nucleated PMNs retained relatively round cell shapes, PMN_cyt_s were more elongated and exhibited reduced cell roundness (Fig. 1m, n). Together, these data support a confinement-associated mechanism linking Ca^2+^ signaling and PAD-related nuclear remodeling to nuclear signal loss and cytoplast formation within the pulmonary microvasculature.

Because the elongated morphology of PMN_cyt_s suggested altered dynamic behavior, we next analyzed their shape changes and motility over time. Whereas nucleated PMNs maintained a relatively stable rounded morphology over time, PMN_cyt_s rapidly alternated between elongation and contraction, indicating increased shape plasticity (Fig. 2a, b and **Extended Data Movies 3, 4**). Although mean perimeter did not differ significantly between PMN_cyt_s and nucleated PMNs, PMN_cyt_s exhibited substantially greater temporal variability in perimeter than nucleated PMNs (Fig. 2c, d). Cell-tracking analysis further showed that PMN_cyt_s moved faster and more directionally than nucleated PMNs (Fig. 2e–g and **Extended Data Movie 3**). These findings suggest that an anuclear PMN-derived state is associated with reduced cellular mechanical constraints, thereby enabling highly deformable and motile behavior in the inflamed lung microvasculature.

Finally, we examined how PMN_cyt_s interact with bacteria during early challenge in the inflamed lung (Fig. 2h–m). After LPS-induced inflammation, GFP-expressing *Pseudomonas aeruginosa* (GFP-PA) was delivered intratracheally, and PMN–bacterium interactions were analyzed by two-photon intravital imaging (Fig. 2h, i). Bacterial exposure did not significantly change PMN_cyt_ frequency relative to PBS-treated controls (Fig. 2j), suggesting that, under these conditions, PMN_cyt_ formation is not acutely induced by bacterial exposure alone. We therefore examined whether pre-existing PMNcyts preferentially engage bacteria. PMN_cyt_s showed a significantly higher fraction of GFP-PA-positive cells, defined as cells with GFP-PA signal within or immediately adjacent to the segmented cell mask, than nucleated PMNs (Fig. 2k, l). PMN_cyt_s also showed an increased fraction of GFP-PA-containing cells, defined as cells with GFP-PA signal detected within the segmented cell mask (Fig. 2k, m). These findings indicate that PMN_cyt_s interact with bacteria more frequently than nucleated PMNs during early PA challenge in vivo. PMN_cyt_s therefore appear to represent motile PMN-derived structures with increased bacteria-engaging capacity in the inflamed lung.

Overall, our data link pulmonary capillary transit to PMN deformation, GsMTx4-sensitive Ca^2+^ responses, PAD-linked nuclear remodeling and the emergence of motile cytoplasts. Because these PMN_cyt_s are associated with increased bacterial engagement during early PA challenge, our findings support a model in which the pulmonary capillary bed functions as a mechanical checkpoint that shapes PMN state and host–microbe interactions (**Extended Data Fig. 1**). More broadly, these findings suggest that organ-specific mechanical environments can shape innate immune cell behavior during infection and inflammation.

## Online Methods

### Mice

Mice were maintained under specific-pathogen-free conditions at the University of Illinois at Chicago (UIC) Research Resources Laboratory and the Louisiana State University Health Sciences Center at Shreveport (LSU Health Shreveport) Animal Resources facility. All animal procedures were performed in accordance with protocols approved by the UIC and LSU Health Shreveport Institutional Animal Care and Use Committees (UIC protocol #24–039 and LSU protocol #P-26–029). Male and female mice aged 10–16 weeks were used. Catchup mice^7^ were obtained from Dr. Gunzer (University Duisburg–Essen) under a material transfer agreement. PMN-specific Salsa6f reporter mice were generated by crossing Catchup mice with Salsa6f mice^8^ (Jackson Laboratory, strain #031968).

### Endotoxemia model and bacterial challenge

For acute lung inflammation, LPS from *Escherichia coli* O111 (L2630, Sigma-Aldrich) was dissolved in PBS and administered once by intraperitoneal injection at 10 mg kg^−1^ body weight. Ca^2+^ imaging was performed 1 h after LPS challenge, and analyses of nuclear dynamics and PMN_cyt_ formation were performed 3 h after LPS challenge. GFP-PA, a GFP-expressing *Pseudomonas aeruginosa* PAO1 strain kindly provided by Dr. Reddy at UIC, was cultured in LB broth (L3522, Sigma-Aldrich) supplemented with ampicillin^9^. Bacterial concentration was determined by plating serial dilutions of overnight cultures on LB–ampicillin agar and counting colony-forming units (CFU). For bacterial challenge, 1 × 10^8^ CFU bacteria were resuspended in 40 μl PBS and delivered intratracheally using a micro-aerosol sprayer (Liquid PenWu Device, BJ-PW-M; Biojane). Lung intravital bacterial imaging was performed 5 h after bacterial challenge. All experiments involving *P. aeruginosa* were conducted under BSL-2 conditions under institutional biosafety approval (UIC protocol #24–017 and LSU protocol #B26–004).

### Pharmacological inhibition

To inhibit mechanosensitive channel activity and PAD activity, GsMTx4 (AB141871; Abcam) and Cl-amidine (506282; Sigma-Aldrich) were used, respectively. GsMTx4 was administered intraperitoneally at 270 μg kg^−1^ at 2 h and 1 h before LPS challenge. Cl-amidine was administered intraperitoneally at 50 mg kg^−1^ at 1 h before LPS challenge.

### Lung intravital imaging

Surgical preparation of the mouse lung and two-photon intravital imaging were performed as described previously^5,6^. Salsa6f-Catchup mice were used for Ca^2+^ imaging, and Catchup mice were used for nuclear imaging. To visualize the pulmonary microvasculature, SeTau647-labelled anti-CD31 antibody (10 μg per mouse; SETA Biomedicals and BioLegend) was injected intravenously via the retro-orbital sinus before surgery. To visualize nuclei, SYTO40 (0.167 mM per mouse; Thermo Fisher Scientific) was administered by the same route before imaging. Time-lapse images were acquired at video rate using a Leica Stellaris 8 Dive multiphoton microscope, a Bruker Ultima In Vivo multiphoton microscope or an Olympus FVMPE-RS multiphoton microscope equipped with a 20–25× objective (Numerical aperture 1.0–1.05). For Ca^2+^ imaging, multicolour images were acquired simultaneously at 940 nm excitation (Emission windows: 515/40 nm for GCaMP, 598/60 nm for tdTomato and 705/90 nm for SeTau647). For nuclear and bacterial imaging, multicolour images were acquired simultaneously at 860 nm excitation (Emission windows: 450/70 nm for SYTO40, 525/50 nm for GFP, 595/60 nm for tdTomato and 708/75 nm for SeTau647).

### Image analysis

Time-lapse images were analyzed using ImageJ, Origin (OriginLab) and custom LabVIEW programs (National Instruments). Respiratory motion artifacts were corrected with the CoVSTii program^6^ using the lung structural signal from SeTau647-labelled anti-CD31 antibody as the reference channel. Ratiometric Ca^2+^ signals were calculated as the GCaMP/tdTomato ratio. Ca^2+^ intensity and cell perimeter were quantified after time-series tracking of individual PMNs. Correlation coefficients between Ca^2+^ intensity and cell perimeter were calculated from paired time-series measurements for each tracked cell. Fisher’s z-transformed values were used for statistical comparison of correlation coefficients between groups. PMNs and PMN_cyt_s were classified using a threshold derived from multi-Gaussian model fitting of SYTO40 nuclear fluorescence intensity distributions. Cellular and nuclear morphologies were quantified from segmentation masks. Cell roundness was calculated as the inverse aspect ratio (minor axis divided by major axis), with higher values indicating a more circular cell shape. Nuclear solidity was calculated as nuclear area divided by convex hull area, with higher values indicating a more compact and less-lobulated nuclear morphology. For cell migration analysis, centroid coordinates were extracted from time-lapse images and used to quantify velocity, total path length and net displacement. The directionality index was calculated as net displacement divided by total path length^6^. For bacterial association analysis, cells were classified as GFP-PA-positive when GFP-PA signal was detected within the segmented cell mask or within a predefined 2 μm pericellular region adjacent to the mask. Cells were classified as GFP-PA-containing when GFP-PA signal was detected within the segmented cell mask.

### Statistics

Statistical analyses were performed using Origin (OriginLab). Individual cells or tracked cells were used as observational units for statistical comparisons. Measurements were obtained from cells within multiple fields of view from independent mice. For time-lapse analyses, repeated measurements from the same tracked cell were used to quantify temporal variability or migration parameters. Two-group comparisons were performed using unpaired two-tailed Student’s t-tests. Comparisons among more than two groups were performed using one-way ANOVA with Tukey’s multiple-comparison test where appropriate. The statistical test used for each comparison, together with the numbers of mice, FOVs and cells, is indicated in the relevant figure legends. A P value < 0.05 was considered statistically significant.

## Supplementary Material

Supplementary Files

This is a list of supplementary files associated with this preprint. Click to download.

- ExtendedMovie4.mp4
- ExtendedMovie3.mp4
- ExtendedMovie1.mp4
- ExtendedMovie2.mp4
- ExtendedDataFiguresandFigureLegends.docx

## Figures and Tables

**Figure 1:**
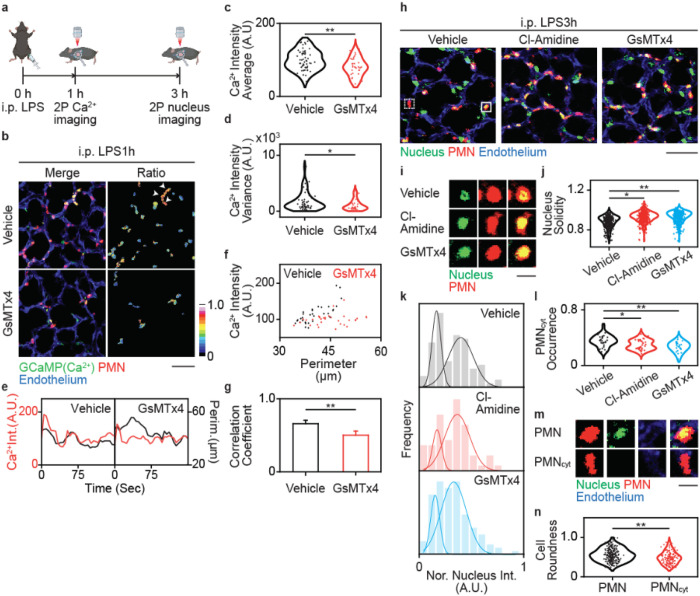
Pulmonary capillary confinement is associated with GsMTx4-sensitive Ca^2+^ responses and PAD-linked PMN nuclear remodeling. **a**, Experimental scheme. **b**, Representative two-photon images and ratiometric Ca^2+^ images of PMNs in the pulmonary microvasculature in vehicle- and GsMTx4-treated mice. Scale bar, 50 μm. **c**,**d**, Quantification of mean Ca^2+^ signal intensity (**c**) and Ca^2+^ signal variance (**d**) in individual PMNs. **e**, Representative time series showing changes in cell perimeter (black) and Ca^2+^ intensity (red) during capillary transit. **f**, Representative scatter plot showing the relationship between Ca^2+^ intensity and cell perimeter. **g**, Comparison of correlation coefficients between Ca^2+^ intensity and cell perimeter. Data are presented as the mean ± SEM. For **c**, **d**, **f**, **g**, *n* = 3 mice, 14–28 fields of view (FOVs) and 34–57 cells. **h**, Representative two-photon images of PMNs, nuclei and pulmonary microvessels 3 h after LPS challenge in vehicle-, Cl-amidine- or GsMTx4-treated mice. Solid boxes indicate nucleated PMNs and dashed boxes indicate anuclear PMN-derived cytoplasts (PMN_cyt_s). Scale bar, 50 μm. **i**, **j**, Representative images of PMN nuclear morphology (**i**) and quantification of nuclear solidity (**j**) under the indicated conditions. Scale bar, 10 μm. **k**, Normalized histogram of nuclear fluorescence intensity. **l**, Quantification of PMN_cyt_ frequency after LPS challenge in vehicle-, Cl-amidine- or GsMTx4-treated mice. **m**, Enlarged views of the boxed regions in **h**. Scale bar, 10 μm. **n**, Quantification of cell roundness in PMNs and PMN_cyt_s. For **j**, **l**, **n**, *n* = 3 mice, 14–34 FOVs and 419–552 cells. *P < 0.05, **P < 0.01; n.s., not significant. Statistical analyses were performed using unpaired two-tailed Student’s *t*-tests (**c**, **d**, **g**, **n**) or one-way ANOVA (**j**, **l**).

**Figure 2:**
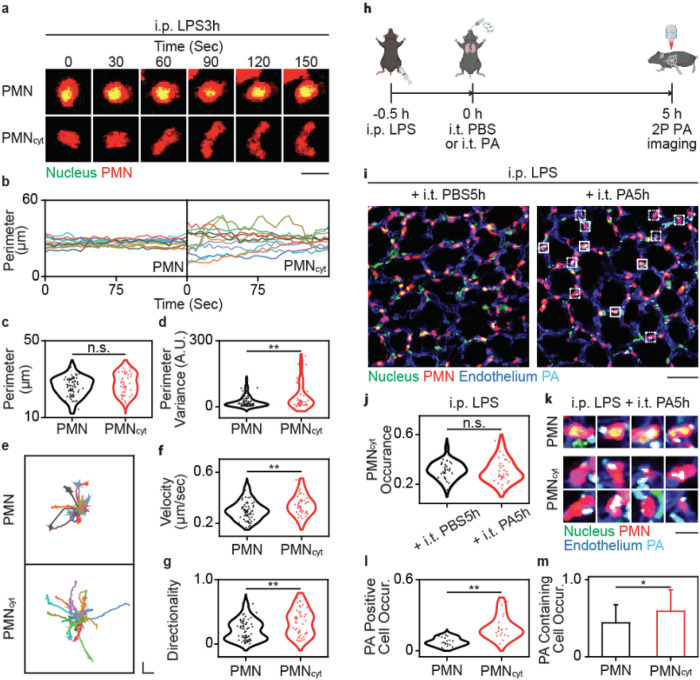
Motile PMN-derived cytoplasts preferentially engage bacteria during early pulmonary infection. **a**, Representative time-lapse images of PMNs and PMN_cyt_s. Scale bar, 10 μm. **b**, Time-dependent changes in cell perimeter in representative PMNs (left) and PMN_cyt_s (right) 3 h after LPS challenge. **c**, Mean cell perimeter in PMNs and PMN_cyt_s. **d**, Temporal variability in cell perimeter, quantified as variance. **e**, Migration tracks of PMNs and PMN_cyt_s. **f**, Quantification of migration velocity. **g**, Quantification of directionality. Directionality was defined as net displacement divided by total path length. For **c**, **d**, **f**, **g**, *n* = 3 mice, 14–28 FOVs and 42–74 cells; for **b**, **e**, 11–24 cells. **h**, Experimental scheme. **i**, Representative intravital images of PMNs, nuclei, pulmonary microvessels and GFP-expressing *Pseudomonas aeruginosa* (GFP-PA) 5 h after infection. Scale bar, 50 μm. **j**, Quantification of PMN_cyt_ frequency after PBS or PA administration. **k**, Enlarged images of PMNs and PMN_cyt_s associated with GFP-PA. Scale bar, 10 μm. **l**, Fraction of GFP-PA-positive PMNs and PMN_cyt_s. **m**, Fraction of GFP-PA-containing PMNs and PMN_cyt_s. Data are presented as the mean ± SD. For **j**, **l**, **m**, *n* = 3 mice and 15–35 FOVs. *P < 0.05, **P < 0.01; n.s., not significant. Statistical analyses were performed using unpaired two-tailed Student’s *t*-tests (**c**, **d**, **f**, **g**, **j**, **l**, **m**).

## Data Availability

The data supporting the findings of this study are available from the corresponding author upon reasonable request.

## Code availability

The custom image-stabilization code (CoVSTii program) is available from the corresponding author upon reasonable request.
